# Informal employment and poor self-perceived health in Latin America and the Caribbean: a gender-based comparison between countries and welfare states in a pooled analysis of 176,786 workers

**DOI:** 10.1186/s12992-021-00792-3

**Published:** 2021-12-05

**Authors:** Mireia Utzet, Ferran Botías, Michael Silva-Peñaherrera, Aurelio Tobías, Fernando G. Benavides

**Affiliations:** 1 Center for research in Occupational Health (Cisal), Universitat Pompeu Fabra (UPF)-IMIM PSMar, CIBER de Epidemilogía y Salud Pública, Barcelona, Spain; 2 Center for Research in Occupational Health, Edificio PRBB, Doctor Aiguader, 88, 08003 Barcelona, Spain; 3 Institute of Environmental Assessment and water Reseach, Spanish Council for Scientific Research, Barcelona, Spain; 4 School of Tropical Medicine and Global Health, Nagasali University, Nagasaki, Spain

**Keywords:** Occupational health, Working conditions, Employment conditions, Welfare state

## Abstract

**Background:**

More than half of the working population in Latin American and Caribbean (LAC) countries is engaged in informal employment. The few previous studies indicate that this employment condition could have negative consequences for workers’ health. The aim of the present study was to estimate the association between self-perceived health and informality in LAC countries according to gender and welfare state type.

**Methods:**

The cross-sectional study based on different working conditions and health national surveys was carried out in 13 LAC countries between 2012 and 2018. A sample of 176,786 workers was selected from these surveys. The association between health and informality was estimated using Poisson regression. Finally, a random effects meta-analysis was carried out by country. All results were stratified by sex and type of welfare state (statalist or familialist).

**Results:**

Informal workers reported significantly worse health than formal workers, for both women (1.28 [95% CI 1.14-1.43]) and men (1.30 [1.12-1.50]). This difference was broader and more significant in countries with statalist welfare state regimes, among both women (1.40 [1.22-1.60]) and men (1.51 [1.30-1.74]), than in familialist regime countries (1.19 [1.03-1.38] and 1.24 [1.03-1.49], respectively).

**Conclusions:**

This study provides strong evidence of the association between informal employment and worker health. Welfare states appear to have a modifying effect on this association. The transition from the informal to the formal labour market in LAC is essential to improving the health of the population.

**Supplementary Information:**

The online version contains supplementary material available at 10.1186/s12992-021-00792-3.

## Introduction

Paid work is the principal income source for the overwhelming majority of people.[1] Work and employment are fundamental social determinants of health that can have both health-damaging and health-enhancing effects.[2] During the last few decades, the scientific interest in different forms of employment conditions and their association with health has grown rapidly,[3] though much is still unknown. Informal employment is one of the most widespread forms of employment in the world, especially in low- and middle-income countries[4]. Although there is literature on the subject[5, 6], the mechanisms and total contribution of this form of employment on worker´s health is still under study[7].

Informal employment is understood as a labour relationship in which the arrangement is not regulated, and workers are not protected by labour regulations or social security benefits. It can be found in both formal and informal economic sectors.[8] This form of employment is characterised by high levels of precariousness, a lack of security that increases the vulnerability of workers, low income, arbitrary labour relations, lack of effective labour representation, and difficulty developing a professional career.[9] The lack of social security reduces access to health care services,[10] and informal employment has been related to unfavourable health outcomes, such as self-perceived health and mental health.[4, 11, 12] In most Latin American and Caribbean (LAC) countries, only formal workers have access to national health systems and social protection benefits.[13] In the last two decades, LAC countries have developed an approach to address health-system reforms, which combine demand-side changes to alleviate poverty and comprehensive primary health care to extend service access.[14].

In LAC countries, informal employment is one of the most extensive forms of employment. Approximately 50% of the non-agricultural working population, comprising roughly 140 million people, have informal employment.[15] Although the proportion of informal employment has slightly decreased in the last two decades, this trend has stagnated since 2015.[16] Women, young people, lower social classes, and people with low levels of education are the most affected by this form of employment.[17] In addition, these workers are more likely to be exposed to poor working conditions and are engaged in low-productivity manual jobs. In the case of women, they may be pressured to work because of their care responsibilities and family constraints, and informal employment is most likely the only option they have to participate in the labour market. Women in informal employment have significantly worse self-perceived and mental health than men.[11].

Welfare states set parameters for the income redistribution programmes that generate social policy to satisfy the basic needs of the population, social protections, health services, pensions, and other worker benefits.[18] Welfare state regimes could greatly influence health[19] and are closely related to labour market regulations and social policies.[20] Thus, they may buffer the relationship between employment and health because the degrees of social protection can reduce the impact of poor conditions at work in population health[21] due to improved employment conditions.

In 2008, Martínez Franzoni[22] analysed the social welfare patterns in LAC from the perspective of gender in four dimensions (i) labour commodification, that is the labour market’s ability to provide decent employment; (ii) welfare decommodification, the ability to guarantee the population’s well-being through redistributive policies without labour market involvement; (iii) welfare familisation, the volume of unpaid care work within families; and (iv) regime performance, the effectiveness of public expenditure and resource allocation. Based on these dimensions, she proposed a typology of three regimes: productivist, protectionist, and familialist (which includes the subgroups familialist and highly familialist). In a productivist regime (e.g., Argentina and Chile), welfare decommodification relies on individual income. In a protectionist regime (e.g., Brazil, Costa Rica, Mexico, Panama, and Uruguay), the state presence is strongest, and there are higher levels of welfare decommodification. Both state regime groups have high levels of labour commodification. In contrast, in a familialist regime (e.g., Colombia, Ecuador, El Salvador, Guatemala, Peru, Dominican Republic, Venezuela) and highly familialist (e.g., Bolivia, Honduras, Nicaragua, and Paraguay), the welfare is mainly provided by community and family arrangements. In this regime, social policies are weak or inexistent, and informal employment accounts for most of the labour market.[12].

The scientific literature analysing health differences and inequities between countries in relation to welfare state regimes is almost limited to high-income countries and the Esping-Andersen typology.[23] Thus, with few exceptions, almost no studies have assessed health inequities in relation to welfare state regimes in low-income countries. The aim of the present study was to estimate the association between self-perceived health (SPH) and informality and to assess whether patterns of this association differ by welfare state regime in 13 LAC countries.

## Methods

The study was based on the most recent national health surveys or surveys on working conditions from a representative sample of LAC countries. We pooled data from countries in which national surveys were carried out since 2012 and included variables that measured SPH and formal or informal employment. We only found data from 13 countries: Argentina, Brazil, Chile, Colombia, Costa Rica, Ecuador, El Salvador, Guatemala, Honduras, Nicaragua, Mexico, Panama, and Peru. In all surveys, questionnaires were administered by personal interviews at the respondent’s home. Participation was voluntary and confidential. The source, year, and sample size of each national survey are described in Supplementary Table S1.

The present study included only workers older than 18 years of age who had been engaged in some form of paid work for at least 1 h during the week preceding the interview. Agricultural and military workers were excluded because of how their characteristics differ from the standard working population.[8] The final sample included pooled data from 176,786 workers. A description of the sample characteristics by welfare state, country, and sex is presented in Table S2.

Worker health status was addressed in each national survey based on a self-assessment of health status. The original Likert scales were dichotomised into ‘good health’ and ‘poor health’. The specific national questions, original scales, and dichotomisations are shown in Table S1. The main independent variable was the type of employment, which was captured by questions on the coverage or contribution to a public health/pension plan/insurance (see Table S1). Among employees, such coverage is considered a proxy of informal employment, with the lack of it being equivalent to the category ‘informal employment’.[8] Mexico’s survey is the exception because it directly asks about the type of employment, including the category informal employment.

Countries were grouped according to an adaptation of the above-mentioned Martínez Franzoni classification. The welfare regimes were grouped into two groups: statalist or familialist. ‘Statalist regime’ brings together the state productivist and state protectionist regimes, which share the characteristics of involving high levels of labour commodification, and of generating social policy from the state, regardless of the level of welfare decommodification. ‘Familialist regime’ includes the familialist and highly familialist countries, where the state does not generate social policy and the family that bears the burden of social support. A list of selected labour indicators by country and gender, grouped by welfare state (Statalist and Familialist) is presented in table S3. As it can be seen in the table, countries have similar labour commodification characteristics within regimes, especially informality rates.

Other socio-demographic variables were included in the analysis, including age (≤24 years, from 25 to 44 years, from 45 to 64 years, and >65 years), education level (low, medium, and high), and occupational category, according to the nine major occupational categories of the International Standard Classification of Occupations (ISCO)[24] collapsed into skilled non-manual (managers, professionals, technicians, and associate professionals), non-skilled non-manual (clerical support workers, service and sales workers), skilled manual (skilled agricultural, forestry and fishery workers, craft and related trades workers, plant and machine operators, and assemblers), and non-skilled manual (elementary occupations).

### Statistical analysis

A two-stage approach was used to analyse pooled cross-sectional data. First, each variable of interest was described in terms of proportions and 95% confidence intervals (CIs) separately by sex and country. The crude prevalence of poor SPH was estimated by type of employment, age, education level, and occupational social class, and stratified by sex and country. Adjusted prevalence ratios (aPRs) between poor SPH and informal/formal employment were estimated for sex and country using Poisson regression with robust variance[25], which is usually preferable to other regression models because it provides unbiased estimates [26]. The final model was adjusted for age, education level, and occupational category. In all the analyses formal workers were the reference category and were stratified by sex and welfare state type.

In a second stage, random-effects meta-analysis[27] was used to pool the aPRs from all countries where the survey was carried out. The heterogeneity of the meta-analysis was quantified by the I^2^ index, which represents the percentage of variation across countries that is due to heterogeneity rather than chance. I^2^ values >75% indicate substantial heterogeneity.[28] Seeking to reduce the possible heterogeneity, a subgroup meta-analysis was carried out according to the type of welfare state. The meta-analyses were also stratified by sex. Statistical analyses were conducted in Stata software, release 16 (StataCorp LLC, College Station, TX, 2019).

## Results

Informal workers (Table 1) reported significantly worse health than formal workers in all countries, except for Guatemala and Nicaragua, and for both men and women except for women in Costa Rica and Honduras, and men in Panama. In addition, a gradient was observed for age, education level, and occupational categories; the older workers, the lower level of education, and the lower occupational category reported the worse SPH status for both men and women in all countries.

**Table 1:** Prevalence of poor self-perceived health by various factors according to welfare state, country, and sex

			Statalist countries						Familialist countries						
			**Argentina**	**Brazil**	**Chile**	**Costa Rica**	**Mexico**	**Panama**	**Colombia**	**Ecuador**	**El Salvador**	**Guatemala**	**Honduras**	**Nicaragua**	**Perú**
			**2018**	**2013**	**(2015-16)**	**2018**	**2012**	**2018**	**2017**	**2012**	**2018**	**2018**	**2018**	**2018**	**2015**
**Women**															
	**Informality**														
		Formal	14.9 (13.4-16.5)	16.0 (15.1-17.0)	20.6 (16.2-25.5)	26.0 (19-34.2)	19.7 (14.7-25.6)	22.8 (18-28.2)	10.7 (9.2-12.4)	31.1 (29.0-33.2)	17.8 (11-26.5)	15.0 (7.4-26.2)	35.2 (24-47.7)	32.2 (24.5-40.9)	34.1 (28.7-39.8)
		Informal	24.1 (21.1-27.4)	30.5 (29.6-31.4)	37.9 (30.7-45.6)	30.0 (22.5-38.4)	30.8 (23.3-39.1)	38.3 (28.4-49.1)	23.8 (22-25.6)	42.8 (41.3-44.3)	33.6 (29.1-38.3)	19.9 (15.9-24.6)	44.3 (39.1-49.5)	46.9 (41.4-52.5)	47.3 (44.1-50.5)
	**Age, years**														
		< 25	11.9 (8.2-16.5)	14.5 (13.2-16.0)	4.5 (1.0-13.1)	11.8 (3.9-26.5)	10.7 (4.6-20.8)	14.0 (5.1-29.3)	10.4 (7.8-13.4)	27.1 (24.6-29.8)	11.6 (5.3-21.6)	15.3 (9.6-22.9)	35.4 (24.2-48.1)	27.5 (17.7-39.4)	29.3 (24.3-34.6)
		25-44	14.4 (12.7-16.3)	21.1 (20.3-22.0)	22.7 (18.4-27.5)	21.1 (14.4-29.3)	20.6 (15.5-26.5)	27.8 (21.2-35.1)	13.7 (12.1-15.3)	37.0 (35.4-38.6)	26.2 (20.9-32.1)	15.3 (10.8-20.7)	38.6 (33-44.3)	41.7 (35.6-48.1)	39.0 (35-43)
		45-64	23.0 (20.4-25.7)	36.7 (35.3-38.2)	37.9 (32.5-43.6)	41.1 (31.6-51.1)	38.0 (27.4-49.6)	38.8 (28.4-50.1)	27.6 (25-30.3)	48.3 (45.9-50.7)	35.8 (28.4-43.8)	33.4 (24.3-43.6)	56.3 (48.5-63.8)	59.1 (50.4-67.3)	55.1 (49-61)
		>65	28.7 (22.2-36)	45.3 (39.9-50.8)	39.1 (23.4-56.7)	45.6 (25.2-67.2)	85.7 (49.9-98.4)	71.8 (40.3-92.1)	42 (33.9-50.4)	61.4 (53.9-68.5)	51.8 (38.7-64.8)	35.8 (18.7-56.2)	78.3 (58.6-91.3)	70.9 (51.9-85.5)	87.8 (79.4-93.6)
	**Education level**														
		Low	29.0 (25.7-32.4)	41.1 (39.7-42.6)	36.4 (29.3-44.1)	34.6 (24.5-45.8)	44.3 (34.3-54.7)	55.2 (35.7-73.6)	34.1 (30.7-37.6)	48.0 (46.1-49.9)	39.3 (32.3-46.6)	22.5 (16.8-29.1)	55.4 (48.3-62.4)	51.5 (44.8-58.2)	64.5 (59.2-69.6)
		Intermediate	16.4 (14.3-18.7)	21.9 (20.9-23.0)	26.8 (22.1-31.8)	25.5 (18.5-33.6)	19.6 (14.3-25.8)	24.9 (18.6-32.2)	18.5 (16.5-20.6)	39.3 (37.4-41.3)	24.8 (20.1-30.1)	17.2 (11.8-23.8)	31.0 (24.6-37.9)	37.1 (29.4-45.3)	39.7 (35.8-43.8)
		High	8.7 (7.1-10.6)	12.9 (12.0-13.8)	19.3 (13.9-25.7)	20.5 (11.6-32.5)	9.9 (4.5-18.4)	25.3 (18.1-33.8)	10.3 (8.8-11.9)	26.1 (23.8-28.5)	24.3 (14.6-36.5)	12.4 (5.9-22.3)	19.8 (11.1-31.6)	31.7 (22.4-42.3)	32.8 (27.9-38)
	**Occupational category**														
		Skilled non-manual		14.5 (13.4-15.6)	20.0 (14.5-26.5)	14.2 (7.2-24.6)	6.7 (1.9-16.7)	24.9 (17.2-34)	10.6 (8.7-12.8)	25.8 (22.9-28.9)	35.9 (18-57.4)	13.5 (5.6-26.4)	36.6 (24.1-50.6)	30.0 (18.6-43.7)	28.1 (22.2-34.7)
		Non-skilled non-manual		23.0 (22.0-24.1)	29.0 (22.8-35.9)	28.4 (23-34.3)	16.3 (7.6-29.3)	23.6 (18.3-29.7)	20.1 (18.4-21.9)	39.8 (37.8-41.7)	26.9 (22.9-31.2)	16.1 (12-20.9)	42.9 (36-50)	36.7 (27.9-46.3)	41.5 (37.6-45.3)
		Skilled manual		36.1 (33.8-38.4)	29.4 (16.0-46.3)	32.3 (20.1-46.6)	19.0 (11.8-28.4)	43.6 (25.3-63.3)	28.3 (24.1-32.7)	42.3 (39.9-44.7)	33.3 (25.9-41.5)	27.3 (18.5-37.7)	43.9 (35.8-52.3)	45.3 (38.4-52.3)	52.3 (43.1-61.5)
		Non-skilled manual		36.4 (34.8-38.0)	31.4 (23.9-39.7)	27.6 (15.5-42.9)	33.1 (26.2-40.6)	35.8 (22.1-51.5)	19.1 (11.4-29.3)	43.7 (41.3-46.1)	41.3 (32.4-50.6)	13.4 (5.5-26.5)	44.6 (32.7-57)	46 (37-55.2)	57.8 (52-63.5)
**Men**															
	**Informality**														
		Formal	12.1 (10.7-13.6)	13.3 (12.5-14.2)	9.6 (6.8-13.1)	19.4 (13.7-26.2)	15.0 (11.7-18.8)	22.2 (17.3-27.9)	7.2 (6.2-8.5)	28.7 (27.1-30.2)	13.6 (6.9-23.3)	28.0 (18.8-38.9)	25.2 (17.4-34.4)	44.8 (35.2-54.7)	29.7 (25.6-34)
		Informal	19.8 (17.3-22.4)	26.2 (25.5-26.9)	20.8 (14.6-28.2)	35.6 (26.3-45.9)	34.7 (29.2-40.6)	23.4 (17.7-29.8)	17.7 (16.4-19.1)	35.0 (33.8-36.1)	29.3 (22.6-36.6)	23.8 (19.6-28.3)	46.8 (42.1-51.6)	49.1 (43.8-54.4)	37.6 (34.4-40.8)
	**Age, years**														
		< 25	8.1 (5.4-11.7)	11.6 (10.6-12.7)	6.4 (1.9-15.9)	16.4 (6.1-33.5)	18.5 (12.3-26.2)	11.9 (5.3-22.3)	5.6 (4-7.6)	22.4 (20.7-24.2)	11.7 (3.7-26.9)	18.5 (11.4-27.8)	19.0 (10.6-30.3)	35.5 (23.3-49.2)	20.6 (16.2-25.5)
		25-44	10.5 (9-12.2)	17.5 (16.8-18.2)	9.7 (6.7-13.5)	16.6 (10.6-24.4)	17.0 (13.4-21.1)	16.3 (11.6-21.9)	9.3 (8.2-10.6)	31.3 (30.0-32.5)	21.2 (14.2-29.7)	17.5 (12.9-23)	38.8 (33.2-44.7)	45.1 (38.9-51.4)	29.7 (26.1-33.5)
		45-64	22.8 (20.3-25.5)	33.0 (31.9-34.2)	19.8 (15.7-24.5)	35.9 (26.5-46.2)	36.2 (28.9-44)	32.0 (24.2-40.7)	18.9 (17.1-20.8)	42.5 (40.6-44.4)	26.2 (16.9-37.6)	32.6 (25.2-40.7)	61.4 (54.8-67.7)	60.4 (52.3-68.1)	46.4 (41.1-51.6)
		>65	20.4 (15.2-26.4)	43.4 (39.8-47.1)	35.6 (23.4-49.5)	35.5 (16.6-58.8)	46.4 (29.1-64.5)	57.6 (40-73.8)	35.2 (30.4-40.3)	53.4 (48.6-58.1)	46.0 (28.2-64.6)	49.8 (34-65.7)	75.2 (64.8-83.8)	76 (57.9-88.9)	55.5 (46.7-64.1)
	**Education level**														
		Low	22.1 (19.8-24.5)	32.2 (31.6-32.9)	22.5 (16.6-29.5)	32.1 (24-41.1)	39.2 (31.7-47.1)	38.7 (29.1-48.9)	25.0 (22.8-27.2)	42.9 (41.5-44.3)	32.5 (23.1-43.2)	31.2 (25.6-37.3)	53.7 (48.5-58.8)	58.7 (51.6-65.6)	51.0 (45.3-56.8)
		Intermediate	10.8 (9-12.7)	16.3 (15.5-17.0)	15.7 (12.2-19.8)	18.6 (12.1-26.9)	20.7 (16.8-25)	16.1 (11.9-21.2)	10.7 (9.4-12)	29.5 (28.2-30.9)	23.1 (16.2-31.3)	16.0 (11-22.3)	24.2 (17.3-32.4)	44.0 (36.1-52.1)	33.6 (30.1-37.2)
		High	7.0 (5.2-9.2)	31.8 (30.8-32.8)	6.0 (3.2-10.2)	9.6 (3.5-20.6)	10.9 (6.5-17)	16.6 (9.9-25.4)	5.8 (4.6-7.1)	20.3 (18.2-22.5)	9.9 (3-23.5)	7.5 (2.2-18.6)	27.3 (16.2-41.1)	33.0 (24.9-42)	24.8 (20.7-29.4)
	**Occupational category**														
		Skilled non-manual		12.7 (11.8-13.7)	8.1 (4.6-13.2)	13.9 (6.4-25.6)	18.2 (11.2-27.2)	16.9 (8.6-28.9)	6.3 (4.7-8.2)	20.5 (17.9-23.3)	11.7 (3.1-29.4)	6.7 (2-16.6)	24.1 (14.2-36.7)	38.4 (27.4-50.4)	24.7 (19.8-30.1)
		Non-skilled non-manual		20.3 (19.1-21.5)	7.6 (3.1-15.5)	17.4 (12.1-23.9)	16.2 (8.9-26.2)	25.4 (17.4-34.9)	11.3 (9.6-13.2)	29.6 (27.5-31.7)	32.4 (24.1-41.7)	13.1 (8.3-19.3)	34.0 (25.6-43.3)	45.0 (39.3-50.7)	29.6 (25.1-34.5)
		Skilled manual		25.7 (24.8-26.6)	19.7 (14.9-25.3)	33.7 (25.6-42.6)	28.4 (22.3-35.3)	24.1 (18.9-30)	19.9 (17.9-22)	35.6 (34.3-36.9)	25.5 (18.5-33.6)	30.0 (24.5-35.9)	47.1 (41.6-52.6)	59.9 (49.6-69.5)	40.9 (36.8-45.1)
		Non-skilled manual		30.3 (28.8-31.9)	23.1 (16.2-31.4)	18.9 (10.1-31.2)	22.5 (18.2-27.4)	20.1 (11.7-31.2)	13.5 (11.7-15.4)	38.3 (36.5-40.1)	32.7 (16.6-52.8)	23.4 (11.7-39.3)	45.6 (34.5-57.1)	39.7 (28.3-52.1)	42.8 (35.6-50.2)

Data are given as prevalence (95% confidence interval). *Prevalence of informality adjusted by age, education level, and social occupational class

Informal employment was significantly associated with poor SPH among women in most countries with a statalist welfare state (Table 2), except for Costa Rica and Mexico. There was no association between informal employment and poor self-perceived health in most countries with a familialist welfare state, except for Colombia, Ecuador, and El Salvador. Among men, informal employment was significantly associated with poor SPH in both statalist (except for Chile and Panama) and familialist countries, except for Guatemala, where informal employment appears to be protective, and Peru, where it is not significant.

**Table 2:** Crude and adjusted prevalence ratios between health and informality by welfare state, country, and sex

Welfare state	Country (survey year)	Women		Men	
		cPR (95% CI)	aPR (95% CI)*	cPR (95% CI)	aPR (95% CI)*
Statalist	Argentina (2018)	1.62 (1.37-1.91)	1.43 (1.20-1.71)	1.63 (1.36-1.95)	1.52 (1.26-1.84)
	Brazil (2013)	1.92 (1.94-2.00)	1.49 (1.43-1.56)	2.01 (1.92-2.10)	1.56 (1.48-1.64)
	Chile (2015-16)	2.09 (1.51-2.88)	1.90 (1.33-2.70)	2.05 (1.34-3.15)	1.32 (0.82-2.13)
	Costa Rica (2018)	1.15 (0.79-1.68)	0.97 (0.69-1.38)	1.84 (1.20-2.81)	1.71 (1.14-2.56)
	Mexico (2012)	1.56 (1.07-2.28)	1.07 (0.73-1.57)	2.31 (1.73-3.09)	1.93 (1.43-2.60)
	Panama (2018)	1.68 (1.18-2.40)	1.45 (1.01-2.08)	1.05 (0.72-1.53)	0.87 (0.58-1.30)
Familialist	Colombia (2017)	2.23 (1.89-2.62)	1.55 (1.29-1.87)	2.44 (2.08-2.87)	1.59 (1.32-1.93)
	Ecuador (2012)	1.39 (1.29-1.50)	1.17 (1.08-1.28)	1.24 (1.16-1.32)	1.09 (1.02-1.16)
	El Salvador (2018)	1.89 (1.24-2.88)	1.79 (1.06-3.02)	2.16 (1.27-3.66)	1.90 (1.13-3.17)
	Guatemala (2018)	1.33 (0.75-2.35)	1.19 (0.65-2.19)	0.85 (0.56-1.29)	0.62 (0.41-0.93)
	Honduras (2018)	1.26 (0.88-1.79)	0.92 (0.65-1.31)	1.86 (1.32-2.62)	1.57 (1.11-2.21)
	Nicaragua (2018)	1.10 (0.86-1.4)	0.92 (0.70-1.22)	1.45 (1.10-1.92)	1.35 (1.02-1.79)
	Peru (2015)	1.40 (1.17-1.67)	1.14 (0.94-1.39)	1.30 (1.10-1.52)	1.10 (0.92-1.31)

cPR, crude prevalence ratio; aPR, adjusted prevalence ratio. ^*^ Adjusted by age, education level, and occupational category

**Fig. 1:**
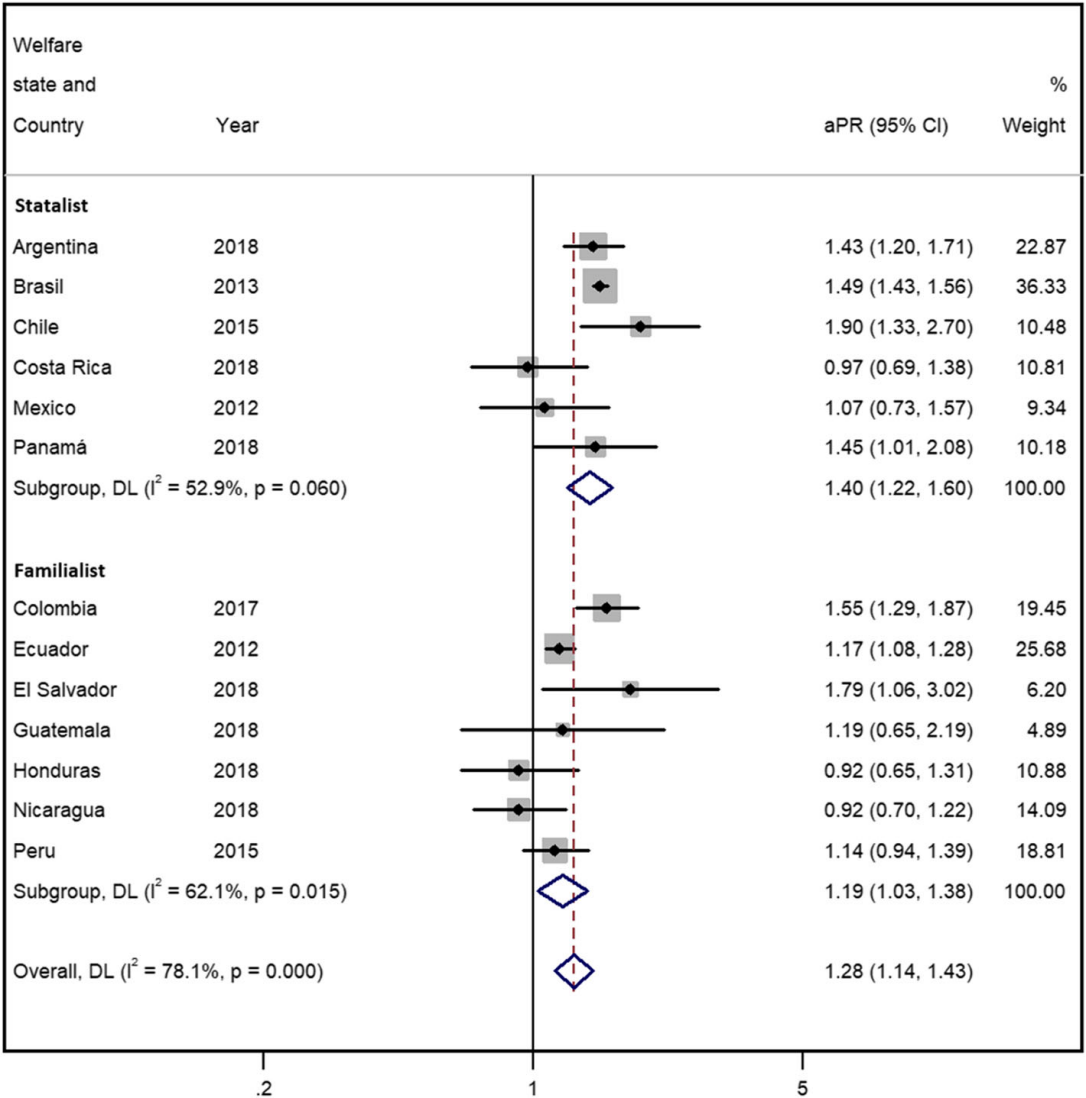
Forest plot of the adjusted prevalence ratio of poor self-perceived health and informal/formal employment among women by welfare state and overall

**Fig. 2:**
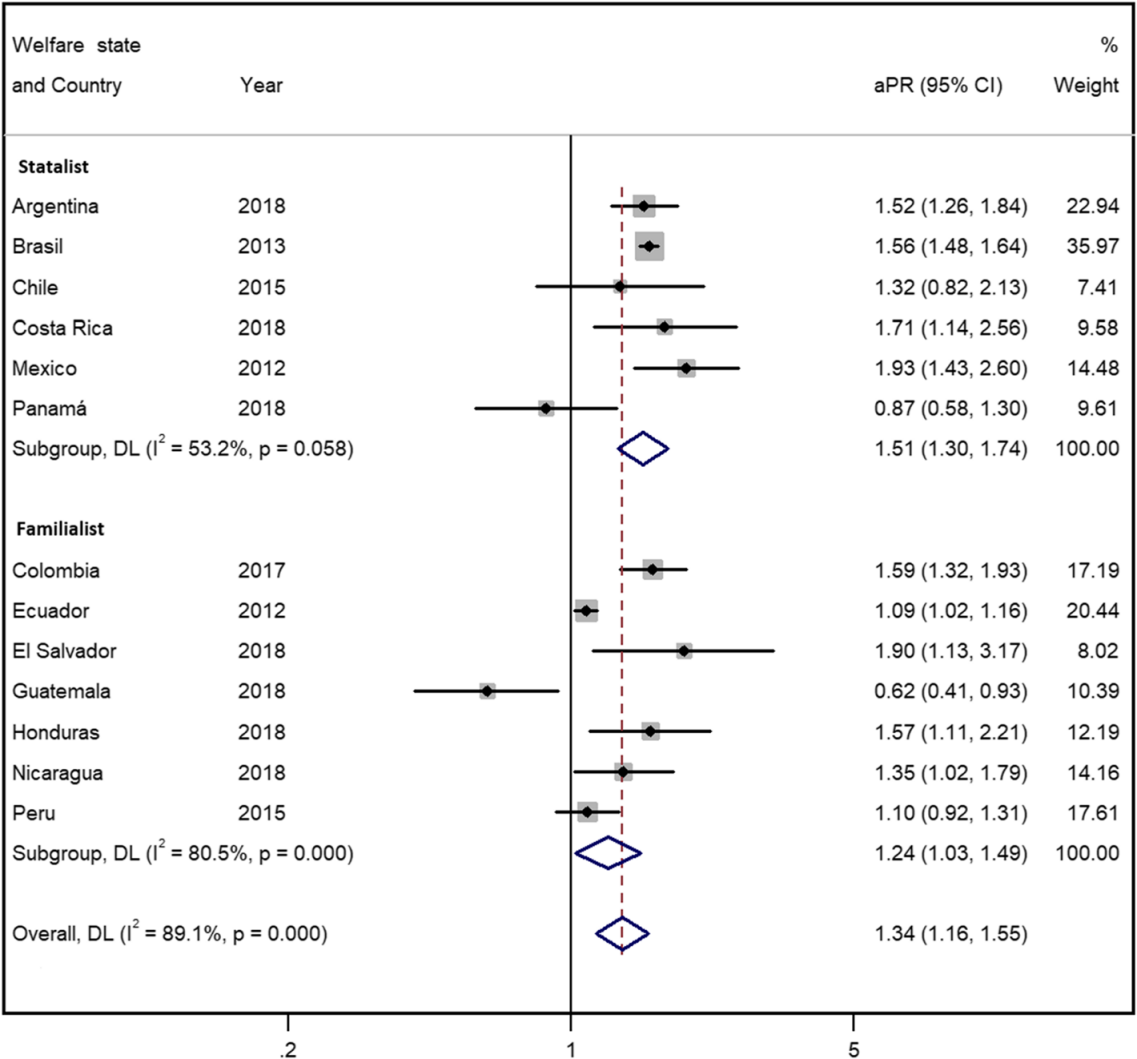
Forest plot of the adjusted prevalence ratio of poor self-perceived health and informal/formal employment among men by welfare state and overall

The results of the gender-stratified meta-analysis including all countries (see Figs. 1 and 2) indicated that informal workers in all countries reported significantly worse health than formal workers (the reference group) for both women and men, with a pooled estimated aPR (95% CI) of 1.28 (1.13-1.43) and 1.30 (1.12-1.50), respectively. The analyses presented significantly high between-countries heterogeneity (I^2^=78.1%, *p* < 0.001 among women, and I^2^=88.6%, *p* < 0.001 among men), i.e. that there are differences underlying the PRs in the countries included.

Based on the type of welfare state, women (Fig. 1) in statalist countries had a higher, though not significant, pooled aPR (95% CI) than women in familialist countries (1.40 [95% CI 1.22-1.60] and 1.19 [1.03-1.38], respectively). Men (Fig. 2) in statalist countries presented a non-significantly higher pooled aPR than men in familialist countries (1.51 [1.30-1.74] and 1.24 [1.03-1.49], respectively). In both men and women, the heterogeneity was lower within both subgroups.

## Discussion

This study found a clear and strong association between informal employment and poor self-perceived health in both men and women and in most of the LAC countries. This association is consistent with previous studies in the Latin America region.[29] The main mechanisms that could explain this relationship include precarious employment, and working and living conditions involving income insecurity, poverty, absence of social protection benefits, and lack of access to health services, which characterise informal employment.[3].

However, this association was broader and more significant in countries with statalist welfare state regimes than in familialist regimes. In this sense, the welfare state, as a complex system of regulation and wealth redistribution, seems to partially buffer this effect on population health, as informality and poor SPH were weaker in familialist countries than in statalist countries, where informality and poor SPH are somewhat lower and the welfare states are more developed.[22] This result seems to contradict other studies, which show that more egalitarian welfare states tend to reduce health inequalities.[30] Yet, none of these studies compared the health of informal and formal workers.

The lower prevalence of poor SPH in the working population of statalist countries is most likely the result of a greater proportion of legally and socially protected workers in the formal labour market. In this regard, formal workers could have access to social and health care services, while informal workers remain unprotected. This greater health disparity between formal and informal workers in the statalist countries could be a consequence of the positive impact that labour policies, social protection and health care have on the formal workers, but it fails to improve the living and working conditions of the large proportion of informal workers and their access to health systems and work-related social security.[31] It seems necessary to set labour policies that consider informal employment, linking the labour market with the public health policies to not only reduce the impact of informal employment, but to promote formal jobs an avoid poor working conditions.

On the other hand, the small health difference between formal and informal workers in familialist countries, especially among women, could be explained by two intertwined hypotheses. First, the weak role of the state in familialist countries. In general, familialist countries have lower gross national income per capita and low social and health expenditures.[15] Consequently, social and health services are poor and, in most of the cases, do not even cover the health needs of the formal workers. In this regard, access to proper health services is mostly determined by the individual income level. Most of the formal workers cannot pay for these services and they are in similar circumstances as informal workers without social and health coverages. Second, the global expansion of labour precariousness and deterioration of working conditions have affected all kinds of employment,[32] regardless of whether they have a contract.[33] This generalised precariousness could explain the lack of association between type of employment and health in familialist countries. As other studies have shown, the impact of specific policies, such as unemployment benefits, seems to be associated with worker health in different types of welfare state.[34] Although labour policies are part of welfare state policies and activities, the classification is broader and includes other types of social policies, it would be interesting in future studies to characterise countries according to labour policies based on employment indicators, in order to estimate the impact of labour policies on the association between informal employment and health.

Regarding gender, women reported a higher prevalence of poor SPH than men in the overwhelming majority of countries in the region, which is consistent with other studies.[35] However, the proportion of women and men with informal employment is similar in almost all countries. Previous studies have found a higher prevalence of informal profiles among women compared to men.[12] We also found that women are not more vulnerable than men to the effects of informality, which was shown in previous studies[35] and reflected in more non-significant associations in familialist countries. In addition to the above considerations, these results could be explained by the social security system being less effective in compensating women than men, because women who have formal employment could continue to experience a precarious situation[12] that negatively impacts their health. In addition, a greater proportion of women in familialist countries could be affected by the “double presence” that involves both the productive and reproductive spheres [11], regardless of the type of employment. This hypothesis will have to be confirmed in future gender-sensitive research, which should include variables that characterise the reproductive sphere and the socioeconomic situation of the family.[11].

This study has some limitations, mainly related to the data sources used. We used the most recent national surveys on working conditions or health surveys available in each country. However, the surveys were not uniform and could slightly differ in the scale used to collect the SPH data. Therefore, comparisons between countries must be made with caution. Furthermore, due to the complexity of measuring and operationalising informality, we used the variable that captures whether the worker has coverage or contributes to a public health/pension plan/insurance to calculate the prevalence of this employment situation. This prevalence represents a proxy of informal employment in the country and could under- or over-estimate the real situation. However, our results for informal employment rates are similar to those reported by the World Bank[36] and those estimated by the International Labour Organisation[37]. Furthermore, this is the most reliable data for measuring the health status of informal workers that the region can currently offer. Official surveys should include variables to better characterise informal employment and its association with health.

In addition, the agricultural working population was excluded from the analysis. Most of the workers in this sector are in an informal situation and likely exposed to worse working and employment conditions than workers in other economic sectors. However, excluding this working population is common practice in most of the studies and a recommendation of the ILO for measuring informality.[8] Moreover, not all LAC countries were included in the study because updated and/or reliable data were not found. However, the analysed sample represents most of the working population in LAC countries. Finally, as in any other cross-sectional study, reverse causality bias could be present, and people with poor health may be more likely to have difficulty finding formal work.

Finally, informal employment has usually been addressed from the economic theory [38], as it is embedded in a given economic context, and is correlated with other factors (such as labour and social protection, health systems, labour market or demographic evolution) that we were not able to include in this paper. However, in our study, welfare state regimes are used to understand how macro-level determinants influence in health, reflecting economic, social structure and power relations within a society. In this regard, welfare state typology is used as an ecological variable to stratify and assess the possible independent effect of all these factors on the association between informality and health.

This study has several strengths. As far as we know, this is the first study in which the patterns of association between informal employment and health were analysed by gender and welfare state in a large representative sample of LAC countries. Second, the results are based on the most updated high-quality data from a representative sample of LAC countries. Data were from national surveys conducted by official institutions in a representative sample of workers in each country, and data collection was via face-to-face interviews at the workers’ homes. Third, the treatment of the data by a meta-analysis allowed us to give a joint measure of association for the countries according to welfare state regimes and sex. It also allowed us to verify that the heterogeneity within each of these regimes is quite high. Notably, the conceptualisation of welfare states implies ideal types, leaving aside the fact that welfare provision varies greatly between countries of the same regime type.[39] In addition, the typology of welfare regime used in this paper was based on a 2008 analysis of economic and social indicators of labour commodification and welfare decommodification. These indicators have likely changed in some countries over the past 12 years. Therefore, more studies are needed to better understand this complex relationship between the health of workers and the policies of the social welfare regime.

## Conclusions

The informal economy embraces a large proportion of the working population in LAC countries, and it is playing an important role in the dynamics of the labour market, wealth generation, and economic activity.[40] In addition, the negative outcomes of informality affect not only the individual, but the state revenues and, therefore, all members of the society.[41] In this regard, reducing informality in the region could be an effective way of ensuring good working and employment conditions, as well as appropriate access to national health systems. In fact, Sustainable Development Goal number 8 of the United Nations 2030 Agenda for Sustainable Development aspires to achieve Decent Work and Economic Growth,[42] for which it is essential to extend rights and opportunities to all workers.

## Supplementary information

**Additional file 1**

## Data Availability

Source by country: Argentina:Instituto Nacional de Estadística y Censos https://www.indec.gob.ar/indec/web/Institucional-Indec-BasesDeDatos-2 Brazil:Instituto Brasileiro de Geografia e Estatística (IBEG) https://ww2.ibge.gov.br/home/estatistica/populacao/pns/2013/default.shtm Chile:Departamento de Epidemiología, ministerio de Salud http://epi.minsal.cl/condiciones-de-uso/Colombia:Dirección Nacional de Estadística http://microdatos.dane.gov.co/index.php/catalog/MICRODATOS Colombia National Quality of Life Survey Costa 2017 https://www.dane.gov.co/index.php/estadisticas-por-tema/pobreza-y-condiciones-de-vida/calidad-de-vida-ecv Costa Rica; El Salvador; Guatemala; Honduras; Nicaragua; Panama:University of Texas, U.S.A.; Universidad Naional de Costa Rica: Available under request Ecuador:Instituto Nacional de Estadística Censos (INEC) http://www.ecuadorencifras.gob.ec/salud-salud-reproductiva-y-nutricion/ México:Institute for Comparative Survey Research http://www.worldvaluessurvey.org/WVSDocumentationWV6.jsp Perú:Instituto Nacional de Salud: Available under requestPuerto Rico:Center of Disease Control and Prevention https://www.cdc.gov/brfss/annual_data/annual_2017.html.
