# Supplementary material for: Informal employment and poor self-perceived health in Latin America and the Caribbean: a gender-based comparison between countries and welfare states in a pooled analysis of 176,786 workers
Source: Global Health. 2021 Dec 5;17:140. doi: 10.1186/s12992-021-00792-3 (PMC8647489; doi:10.1186/s12992-021-00792-3)
Supplement: Supplementary file 1 — Additional file 1 [file 12992_2021_792_MOESM1_ESM.docx]

Supplementary material

**Table S1. Source, year, and sample size of each national survey.**

| **Country** | **Survey** | **Year** | **Sample size**  **(N=176,786)** | **Response Rate** | **Informality** | | | **Self-perceived health** | | |
| --- | --- | --- | --- | --- | --- | --- | --- | --- | --- | --- |
|  |  |  |  |  | **Question** | **Original categories** | **Dichotomisation** | **Question** | **Original scale** | **Dichotomisation** |
| **Argentina** | National Risk Factors Survey | 2018 | 17,457 | 73,0% | Are you associated with…? | 1. Social security, prepaid or emergency medical service; 2. Only public coverage | 1. Formal; 2. Informal | In general, would you say your health is...? | 1. Excellent; 2. Very good; 3. Good; 4. Fair; 5. Poor | 1,2,3: Good health; 4,5: Bad health |
| **Brazil** | National Health Survey | 2013 | 92,168 | 86,0% | Do you have a private, company, or public health plan? | 1. Yes; 2. No | 1. Formal; 2. Informal | In general, how do you rate your health? | 1. Very good; 2. Good; 3. Fair; 4. Bad; 5. Very bad | 1,2: Good health; 3,4,5: Bad health |
| **Chile** | National Quality of Life and Health Survey | 2015-16 | 3,647 | 78,7% | Public health system FONASA or others | 1. Group A; 2. B; 3. C; 4. D; 6. Other; 7. None | 1,2,3,4: Formal; 6,7: Informal | In general, you would say your health is: | 1. Excellent; 2. Very good; 3. Good; 4. Fair; 5. Poor | 1,2: Good health; 3,4,5: Bad health |
| **Colombia** | National Quality of Life Survey | 2017 | 16,812 | 85%% | Are you affiliated with any social security health insurance entity? | 1. Yes; 2. No | 1. Formal; 2. Informal | The health status of "Name" in general is: | 1. Very good; 2. Good; 3. Fair; 4. Very bad | 1,2: Good health; 3,4: Bad health |
| **Ecuador** | National Health and Nutrition Survey | 2011-2013 | 33,552 | 85,8% | You contribute to, are affiliated with, or covered by the IESS Insurance: | 1. General; 2. Voluntary; 3. Farmer; 4. Not affiliated | 1,2,3: Formal; 4. Informal | In general, you would say your health is: | 1. Excellent; 2. Very good; 3. Good; 4. Fair; 5. Poor | 1,2: Good health; 3,4,5: Bad health |
| **Mexico** | World Values Survey: Mexico Questionnaire - 6th Wave | 2012 | 996 | 30,3% | Do you work in...? | 1.Government or public sector; 2. Business or private sector; 3. Private non-profit organisation; 4. Informal sector or self-employed | 1,2,3: Formal; 4. Informal | In general, how would you describe your state of health today? | 1. Very good; 2. Good; 3. Fair; 4. Poor | 1,2: Good health; 3,4: Bad health |
| **Peru** | Working Conditions, Safety and Health in the economically active population | 2015 | 3,122 | 90,0% | Do you have a discount, do you contribute, are you affiliated with, or registered in any retirement system (ONP/AFP)? | 1. Yes; 2. No | 1. Formal; 2. Informal | How do you consider your general state of health? | 1. Very good; 2. Good; 3. Fair; 4. Poor; 5. Very bad | 1,2: Good health; 3,4,5: Bad health |
| **Costa Rica, El Salvador, Guatemala, Honduras, Nicaragua, and Panama** | Second Central American Survey of Working and Health Conditions | 2018 | 9,022 | 26,4% | Are you currently contributing (...) to any retirement, unemployment or disability insurance, or social security or insurance fund? | 1. Yes; 2. No | 1. Formal; 2. Informal | What do you consider your health to be like? | 1. Very good; 2. Good; 3. Fair; 4. Poor; 5. Very bad | 1,2: Good health; 3,4,5: Bad health |

**Table S2.** **Distribution of variables by welfare state, country, and sex**

|  |  |  | **Statalist countries** | | | | | |  | **Familialist countries** | | | | | | |
| --- | --- | --- | --- | --- | --- | --- | --- | --- | --- | --- | --- | --- | --- | --- | --- | --- |
|  |  |  | **Argentina** | **Brazil** | **Chile** | **Costa Rica** | **Mexico** | **Panama** |  | **Colombia** | **Ecuador** | **El Salvador** | **Guatemala** | **Honduras** | **Nicaragua** | **Perú** |
|  |  |  | **2018** | **2013** | **(2015-16)** | **2018** | **2012** | **2018** |  | **2017** | **2012** | **2018** | **2018** | **2018** | **2018** | **2015** |
| **Women** | | |  |  |  |  |  |  |  |  |  |  |  |  |  |  |
|  | **Poor self-perceived health** | | 17.6 (16.2-19.1) | 24.9 (24.2-25.6) | 26.6 (23.4-33.6) | 27.8 (22.5-33.6) | 24.0 (19.7-28.9) | 30.1 (24.6-32.6) |  | 18.2 (16.9-19.5) | 39.0 (37.8-40.2) | 31.6 (27.5-35.8) | 19.4 (15.6-23.6) | 43.2 (38.4-48) | 47.9 (43.2-52.6) | 43.7 (41.0-46.5) |
|  | **Informality** | | 29.3 (27.6-31.1) | 63.5 (62.7-64.3) | 31.8 (27.6-36.3) | 45.8 (39.7-51.9) | 39.0 (33.9-44.4) | 47.0 (40.9-53.4) |  | 57.3 (55.6-58.9) | 62.7 (61.5-64.0) | 87.9 (85.3-90.1) | 88.3 (85.0-91.0) | 87.6 (84.3-90.3) | 72.5 (67.8-76.7) | 73.8 (71.1-76.3) |
|  | **Age, years** | |  |  |  |  |  |  |  |  |  |  |  |  |  |  |
|  |  | < 25 | 11.4 (10.3-12.6) | 15.5 (14.9-16.1) | 10.6 (8.5-13.1) | 11.0 (7.5-15.3) | 16.5 (12.9-20.9) | 16.0 (12-20.8) |  | 13.9 (12.8-15.1) | 16.5 (15.6-17.4) | 8.7 (6.4-11.5) | 42.1 (37.2-47.1) | 28.2 (24-32.7) | 17.2 (13.9-21) | 22.8 (20.5-25.3) |
|  |  | 25-44 | 50.7 (48.9-52.6) | 53.1 (52.3-53.9) | 51.9 (48.1-55.6) | 51.2 (45-57.3) | 60.4 (55.0-65.5) | 52.2 (46.1-58.1) |  | 53.8 (52.1-55.4) | 54.2 (53.0-55.5) | 43.1 (38.7-47.5) | 36.1 (31.4-41.1) | 45.4 (40.6-50.3) | 56.5 (51.8-61.1) | 47.8 (45.0-50.7) |
|  |  | 45-64 | 33.1 (31.4-34.9) | 29.6 (28.9-30.4) | 33.8 (30.3-37.5) | 34.9 (29.2-41) | 21.0 (17.0 -25.7) | 29.0 (23.8-34.6) |  | 29.0 (27.5-30.5) | 27.0 (25.9-28.1) | 35.5 (31.4-39.9) | 17.0 (13.5-21) | 22.7 (18.8-27) | 23.3 (19.5-27.5) | 23.0 (20.7-25.6) |
|  |  | >65 | 4.7 (4-5.6) | 1.7 (1.5-1.9) | 3.7 (2.5-5.3) | 3 (1.4-5.7) | 2.1 (1.0-4.4) | 2.9 (1.3-5.4) |  | 3.4 (2.8-4) | 2.3 (1.9-2.6) | 12.7 (9.9-15.9) | 4.8 (3-7.3) | 3.7 (2.2-5.9) | 2.9 (1.6-4.9) | 6.4 (5.1-7.8) |
|  | **Education level** | |  |  |  |  |  |  |  |  |  |  |  |  |  |  |
|  |  | Low | 27.9 (26.3-29.6) | 29.4 (28.6-30.1) | 21.6 (18.5-24.9) | 35.2 (29.4-41.2) | 26.1 (21.7-31.1) | 16.4 (12.3-21.2) |  | 19.9 (18.6-21.3) | 35.0 (33.8-36.2) | 47.1 (42.7-51.6) | 50.3 (45.2-55.3) | 54.1 (49.2-58.9) | 40.7 (36.2-45.4) | 23.7 (21.5-26.1) |
|  |  | Intermediate | 42.5 (40.7-44.4) | 40.2 (39.4-41.0) | 46.8 (43.0-50.7) | 47.1 (41-53.3) | 52.9 (47.5-58.2) | 51.7 (45.7-57.7) |  | 38.7 (37.1-40.4) | 39.4 (38.2-40.6) | 42.9 (38.6-47.4) | 39.1 (34.3-44.1) | 36.7 (32.2-41.5) | 40.2 (35.6-44.8) | 48.2 (45.4-51.0) |
|  |  | High | 29.6 (27.9-31.3) | 30.4 (29.6-31.1) | 31.6 (28.1-35.3) | 17.7 (13.4-22.8) | 21.0 (17.0-25.7) | 31.9 (26.5-37.7) |  | 41.3 (39.7-43) | 25.6 (24.5-26.7) | 9.9 (7.5-12.8) | 10.6 (7.8-14.1) | 9.2 (6.7-12.3) | 19.1 (15.6-23) | 28.1 (25.6-30.7) |
|  | **Occupational category** | |  |  |  |  |  |  |  |  |  |  |  |  |  |  |
|  |  | Skilled non-manual | - | 27.5 (26.7-28.2) | 35.8 (31.7-40.4) | 9.0 (5.9-13) | 13.5 (10.2-17.6) | 17.0 (12.9-21.9) |  | 26 (24.6-27.5) | 15.7 (14.8-16.6) | 4.4 (2.8-6.5) | 7.2 (4.9-10.2) | 6.7 (4.6-9.5) | 10.8 (8.1-14) | 17.9 (15.8-20.1) |
|  |  | Non-skilled non-manual | - | 38.3 (37.5-39.1) | 34.4 (30.3-38.6) | 39.5 (33.6-45.6) | 12.9 (9.7-17.0) | 44.7 (38.8-50.7) |  | 58.3 (56.6-60) | 42.1 (40.9-43.4) | 40.5 (36.2-44.9) | 51.2 (46.2-56.2) | 35.9 (31.4-40.7) | 39.9 (35.4-44.6) | 51.1 (48.2-53.9) |
|  |  | Skilled manual | - | 11.3 (10.7-11.8) | 4.5 (2.9 -6.5) | 23.8 (18.8-29.4) | 24.9 (20.6-29.9) | 19.8 (15.3-24.9) |  | 13 (11.9-14.2) | 19.3 (18.3-20.3) | 45.8 (41.4-50.2) | 32.8 (28.2-37.6) | 36.7 (32.1-41.4) | 30.9 (26.7-35.3) | 8.2 (6.8-9.8) |
|  |  | Non-skilled manual | - | 23.0 (22.3-23.7) | 25.4 (21.7-29.3) | 27.7 (22.4-33.5) | 48.6 (43.3-54.0) | 18.5 (14.2-23.5) |  | 2.7 (2.2-3.3) | 22.9 (21.9-23.9) | 9.3 (6.9-12.1) | 8.8 (6.3-12) | 20.6 (16.9-24.8) | 18.4 (15-22.3) | 22.8 (20.6-25.3) |
| **Men** | | |  |  |  |  |  |  |  |  |  |  |  |  |  |  |
|  | **Poor self-perceived health** | | 14.7 (13.5-16.1) | 21.6 (21.1-22.2) | 14.4 (11.9-17.2) | 25.5 (20.3-31.3) | 22.9 (19.9-26.3) | 22.9 (18.9-27.4) |  | 13.4 (12.5-14.4) | 33.3 (32.4-34.2) | 26.7 (21.1-32.9) | 24.3 (20.5-28.5) | 43.8 (39.6-48.2) | 43.4 (38.7-48.1) | 34.8 (32.3-37.4) |
|  | **Informality** | | 34.3 (32.6-36.1) | 69.3 (68.6-67.1) | 26.7 (22.9-30.9) | 37.7 (31.8-44.0) | 39.8 (36.1-43.6) | 56.2 (51.4-60.8) |  | 59.2 (57.8-60.6) | 62.0 (61.0-62.9) | 83.8 (79.5-87.3) | 88.0 (85.1-90.4) | 85.8 (82.9-88.3) | 76.0 (72.0-79.6) | 62.7 (60.0-65.3) |
|  | **Age, years** | |  |  |  |  |  |  |  |  |  |  |  |  |  |  |
|  |  | < 25 | 13.2 (12-14.4) | 15.8 (15.3-16.4) | 9.3 (7.3-11.7) | 8.5 (5.5-12.6) | 18.1 (15.3-21.2) | 17.0 (13.5-20.9) |  | 13.4 (12.5-14.4) | 19.6 (18.8-20.4) | 11.3 (7.6-16.1) | 28.2 (24.1-32.5) | 23.4 (19.9-27.3) | 20.4 (16.8-24.4) | 20.6 (18.6-22.9) |
|  |  | 25-44 | 48.6 (46.8-50.5) | 50.7 (50.0-51.5) | 48.0 (44.2-51.7) | 44.9 (38.7-51.2) | 54.7 (50.8-58.5) | 45.0 (40.2-49.9) |  | 48.4 (47.1-49.8) | 50.2 (49.3-51.2) | 37.8 (31.5-44.5) | 36.9 (32.5-41.5) | 39.9 (35.7-44.2) | 55 (50.2-59.7) | 44.6 (41.9-47.3) |
|  |  | 45-64 | 31.9 (30.2-33.6) | 30.2 (29.5-30.9) | 38.5 (34.9-42.2) | 37.2 (31.3-43.4) | 23.1 (20.0-26.5) | 32.4 (27.9-37.1) |  | 32.3 (31-33.6) | 26.8 (25.9-17.6) | 30.5 (24.6-37) | 27.7 (23.6-32) | 26.5 (22.8-30.4) | 22 (18.3-26.2) | 26.2 (23.8-28.6) |
|  |  | >65 | 6.3 (5.5-7.3) | 3.2 (3.0-3.5) | 4.2 (2.9-5.9) | 9.4 (6.2-13.5) | 4.1 (2.8-6.0) | 5.6 (3.7-8.2) |  | 5.9 (5.3-6.6) | 3.4 (3.1-3.8) | 20.3 (15.3-26.1) | 7.2 (5.1-9.9) | 10.2 (7.8-13) | 2.5 (1.3-4.4) | 8.7 (7.3-10.2) |
|  | **Education level** | |  |  |  |  |  |  |  |  |  |  |  |  |  |  |
|  |  | Low | 41.3 (39.5-43.1) | 41.6 (40.8-42.3) | 24.5 (21.4-27.9) | 57.5 (51.2-63.6) | 23.3 (20.2-26.7) | 30.0 (25.6-34.6) |  | 29.0 (27.7-30.2) | 39.2 (38.3-40.2) | 49.3 (42.7-56.1) | 58.9 (54.2-63.4) | 66.0 (61.8-70) | 49.3 (44.5-54) | 18.6 (16.6-20.7) |
|  |  | Intermediate | 40.4 (38.7-42.3) | 38.6 (37.9 -39.3) | 41.1 (37.4-44.8) | 33.2 (27.5-39.4) | 55.9 (52.1-59.7) | 55.9 (51-60.7) |  | 42.9 (41.5-44.2) | 44.6 (43.7-45.6) | 42.6 (36.1-49.4) | 33.2 (28.9-37.7) | 29.2 (25.4-33.2) | 35.9 (31.5-40.5) | 52.3 (49.6-55.0) |
|  |  | High | 18.3 (16.9-19.7) | 19.9 (19.3-20.5) | 34.4 (30.9-38.1) | 9.3 (6.1-13.4) | 20.8 (17.9-24.1) | 14.2 (11-17.9) |  | 28.2 (26.9-29.4) | 16.1 (15.5-16.9) | 8.0 (4.9-12.3) | 7.9 (5.6-10.7) | 4.9 (3.2-7) | 14.8 (11.7-18.4) | 29.1 (26.7-31.6) |
|  | **Occupational category** | |  |  |  |  |  |  |  |  |  |  |  |  |  |  |
|  |  | Skilled non-manual | - | 22.5 (21.9-23.1) | 34.4 (30.5-38.5) | 8.1 (5.1-12) | 13.5 (11.1-16.3) | 7.2 (5-10.1) |  | 15.5 (14.5-16.6) | 10.4 (9.8-10.9) | 3.6 (1.7-6.8) | 5.7 (3.8-8.2) | 6.5 (4.6-8.8) | 6.3 (4.3-8.9) | 21.6 (19.4-24.0) |
|  |  | Non-skilled non-manual | - | 20.9 (20.3-21.5) | 15.1 (12.2-18.3) | 12.0 (8.4-16.6) | 10.4 (8.3-13.0) | 13.5 (10.4-17.1) |  | 25.3 (24.1-26.6) | 20.2 (19.4-21.0) | 14.5 (10.3-19.7) | 21.8 (18.1-25.8) | 11.1 (8.6-14) | 13.5 (10.5-17) | 28.5 (26.1-31.1) |
|  |  | Skilled manual | - | 41.4 (40.6-42.1) | 34.9 (31.0-39.0) | 48.7 (42.4-55) | 28.0 (24.7-31.6) | 58.5 (53.6-63.2) |  | 32.2 (30.9-33.6) | 45.3 (44.4-46.3) | 72.0 (65.7-77.7) | 62.4 (57.8-66.8) | 64.1 (59.9-68.2) | 49.6 (44.9-54.4) | 37.3 (34.7-39.9) |
|  |  | Non-skilled manual | - | 15.3 (14.8-15.8) | 15.6 (12.7-18.9) | 31.2 (25.6-37.3) | 48.1 (44.3-51.9) | 20.8 (17-25) |  | 26.9 (25.7-28.2) | 24.4 (23.3-25.0) | 9.9 (6.4-14.5) | 10.1 (7.5-13.2) | 18.4 (15.2-21.9) | 30.6 (26.3-35.1) | 12.6 (11.0-14.5) |

Data are given as % distribution (95% confidence interval).

Table S3. Labour commodification indicators (2018) by country, sex and welfare state.

|  |  | Employment-to-population ratio (%) (a) | | Unemployment rate (%) (a) | | | Informality rate (%)* (b) | | | Share of salaried workers with right to pensions when retired* (b) | | | | GNI per capita, PPP (c) |
| --- | --- | --- | --- | --- | --- | --- | --- | --- | --- | --- | --- | --- | --- | --- |
|  |  | Women | Men | Women | Men | Women | | Men | Women | | Men | |  | |
| Statalist | Argentina | 45.5 | 66.8 | 10.5 | 8.2 | 41.4 | | 35.2 | 66.4 | | 73.6 | | 22470 | |
|  | Brazil | 46.6 | 66.2 | 14.2 | 10.9 | 42.2 | | 40.5 | 79.2 | | 81.9 | | 14600 | |
|  | Chile | 47.5 | 68.9 | 6.7 | 8.0 | 33.7 | | 28.1 | 84.4 | | 88.6 | | 23620 | |
|  | Costa Rica | 42.2 | 70.4 | 12.2 | 8.0 | 42.5 | | 33.4 | 69.1 | | 77.4 | | 19870 | |
|  | Mexico | 42.6 | 76.1 | 3.4 | 3.2 | 49.4 | | 38.1 | 41.8 | | 42.2 | | 19710 | |
|  | Panama | 50.7 | 77.5 | 5.0 | 3.2 | 41.5 | | 39.4 |  | |  | | 29530 | |
| Familialist | Colombia | 50.5 | 75.1 | 11.8 | 7.1 | 56.6 | | 54.6 | 68.8 | | 72.3 | | 14480 | |
|  | Ecuador | 52.7 | 78.8 | 4.4 | 2.9 | 66.8 | | 55.0 | 66.7 | | 55.9 | | 11530 | |
|  | El Salvador | 43.4 | 72.4 | 4.2 | 3.8 | 60.4 | | 45.6 | 54.8 | | 52.1 | | 8320 | |
|  | Guatemala | 38.7 | 84.7 | 3.5 | 1.9 | 67.7 | | 54.8 | 40.5 | | 31.5 | | 8490 | |
|  | Honduras | 48.1 | 82.0 | 7.2 | 4.7 | 66.7 | | 54.8 | 50.4 | | 34.8 | | 5360 | |
|  | Nicaragua | 47.4 | 80.0 | 5.3 | 5.2 | 70.4 | | 51.4 | 58.1 | | 41 | | 5570 | |
|  | Peru | 67.9 | 82.4 | 3.4 | 3.0 | 66.6 | | 52.3 | 51.2 | | 56.6 | | 12430 | |
| * among 25-64 years old workers. Chile 2017, Guatemala 2014, Nicaragua 2014. | | | | | | | | | |  | |  | |  |

Sources: (a) International Labour Organization (ILO); (b) CEDLAS, The World Bank. Socio-Economic Database for Latin America and the Caribbean (SEDLAC); (c) The World Bank Group. Data Bank.
